# Osmolality Effects on CHO Cell Growth, Cell Volume, Antibody Productivity and Glycosylation

**DOI:** 10.3390/ijms22073290

**Published:** 2021-03-24

**Authors:** Sakhr Alhuthali, Pavlos Kotidis, Cleo Kontoravdi

**Affiliations:** Department of Chemical Engineering, Imperial College London, London SW7 2AZ, UK; s.alhuthali15@imperial.ac.uk (S.A.); p.kotidis17@imperial.ac.uk (P.K.)

**Keywords:** Chinese hamster ovary cells, monoclonal antibody, hyperosmolality, cell volume, antibody glycosylation

## Abstract

The addition of nutrients and accumulation of metabolites in a fed-batch culture of Chinese hamster ovary (CHO) cells leads to an increase in extracellular osmolality in late stage culture. Herein, we explore the effect of osmolality on CHO cell growth, specific monoclonal antibody (mAb) productivity and glycosylation achieved with the addition of NaCl or the supplementation of a commercial feed. Although both methods lead to an increase in specific antibody productivity, they have different effects on cell growth and antibody production. Osmolality modulation using NaCl up to 470 mOsm kg^−1^ had a consistently positive effect on specific antibody productivity and titre. The addition of the commercial feed achieved variable results: specific mAb productivity was increased, yet cell growth rate was significantly compromised at high osmolality values. As a result, Feed C addition to 410 mOsm kg^−1^ was the only condition that achieved a significantly higher mAb titre compared to the control. Additionally, Feed C supplementation resulted in a significant reduction in galactosylated antibody structures. Cell volume was found to be positively correlated to osmolality; however, osmolality alone could not account for observed changes in average cell diameter without considering cell cycle variations. These results help delineate the overall effect of osmolality on titre and highlight the potentially negative effect of overfeeding on cell growth.

## 1. Introduction

Culture osmolality, cell cycle distribution and cell volume of Chinese hamster ovary (CHO) cells have all been shown to correlate with specific recombinant protein productivity, as well as with one another [1,2,3,4,5,6]. The measurement of cell size is typically obtained as part of a cell density and culture viability assessment, and its value often used to calculate the metabolic flux across the cell membrane. Biosynthetic capacity and nutrient exchange depend on cell size [2,7], which, in turn, is influenced by culture osmolality, with hyperosmolar conditions known to lead to increased cell size [8]. The nucleus, mitochondria, endoplasmic reticulum size and protein production and gene expression levels scale with cell size in human and yeast cells [9,10,11].

Lloyd et al. have previously shown that cell size is a determinant of specific protein productivity in four industrially relevant CHO cell lines [12]. Although cells in every cell cycle phase were productive, it was cells in G_2_/M that displayed the highest productivity. Their findings suggest that this was mainly due to the larger cell size in that phase and less because of their cell cycle position. The same observation was made by researchers who expressed human mTOR in CHO cells to enhance manufacturability [13]. However, other studies have reported that cell volume alone is not always sufficient to account for an increase in recombinant protein expression [14], and other factors such as cell culture phase and medium composition strongly affect specific productivity [15,16]. Volume homeostasis is achieved by a balance between the production and consumption of molecules [17]. Each cell is meant to produce or degrade macromolecules at a specific rate to uphold biological functions and either maintain homeostasis or respond to a stimulus. The type and amount of biosynthetic activity may vary significantly between cells, i.e., rapidly dividing versus non-dividing. Cell size is determined by the rate of synthesis and uptake of molecules and their loss by secretion and degradation, which all together can vary with the level of growth factor signalling and gene expression [17,18].

In the context of recombinant protein production, the stationary phase and late stage culture is often considered to be the main production phase. It is accompanied by an increase in the fraction of cells in the G_1_ phase and an increase in the average cell diameter [19]. Attempts have been made to enhance productivity by arresting cells in the cell cycle phase that has the highest productivity through starvation or addition of a small molecule regulator [20,21]. However, late stage culture is also associated with an accumulation of extracellular metabolites, contributing to osmolality increase, which can be exacerbated by overfeeding.

Osmolality has been linked to both cell volume regulation and recombinant protein productivity. Typically, hyperosmotic conditions lead to an increase in cell size [22]. There have been several studies in which, for example, hyperosmolar conditions were introduced to increase recombinant protein titre [23,24]. Interestingly, this occurs despite the negative effect of hyperosmotic conditions on the cell proliferation rate [25]. Nasseri et al. found that both hyperosmolality and 3-methyl adenine treatment were equally effective in increasing the specific productivity of therapeutic protein in different CHO cell lines [26].

In certain studies, the increase in titre was accompanied by varying protein quality, particularly with respect to glycosylation patterns [27], while others reported no impact in this regard [26]. Specifically, hyperosmolality has been found to reduce the proportion of acidic isoforms and sialic acid content in N-linked glycans of a Fc-fusion protein, which can be avoided by betaine addition [28]. Similarly, osmolality increases by 60–120 mOsm kg^−1^ at the stationary phase of a fed-batch culture have been found to lead to the secretion of under-processed glycans [23]. Overall, the effect on recombinant protein glycosylation appears to be dependent on the cell line and extent of osmolality increase [8].

Herein, we investigate the effect of hyperosmolality on CHO cell growth rate, cell volume, specific monoclonal antibody (mAb) productivity and the N-linked glycosylation profile. We use two different additives, a commercial culture feed and NaCl, to increase the level of osmolality, to understand whether variations in cellular behaviour are due to the increase in osmolality alone, the repeated addition of nutrients to the culture and resulting metabolite accumulation, or differences in cell cycle distribution throughout cell culture duration.

## 2. Results and Discussion

### 2.1. Effect of Hyperosmolality on Cell Growth and Metabolism

**Figure 1:**
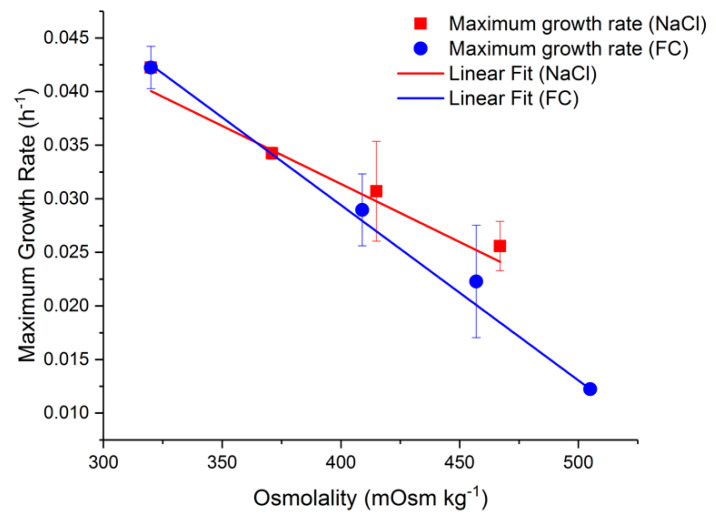
Maximum growth rate of Chinese hamster ovary (CHO) cells as a function of extracellular osmolality. The first point at 320 mOsm kg^−1^ represents the control condition.

We first evaluated the effect of hyperosmolality on the specific cell growth rate and metabolic activity in cultures grown in flasks. Hyperosmolality is typically caused by the repeated addition of feeds and the accumulation of metabolic by-products in fed-batch cultures. The effect of hyperosmolality has previously been studied by adding NaCl to the culture medium [29,30]. However, comparing the NaCl method of osmolality increase to nutrient spiking has not been explored in the literature. Herein, we added either NaCl or Feed C to the culture medium before inoculation to achieve a range of initial osmolality values between 320 and 500 mOsm kg^−1^. As shown in Figure 1, increased osmolality caused a considerable reduction in the maximum specific cell growth rate in both cases. The difference in maximum growth rate under the two types of treatment was not statistically significant (*p* > 0.05).

Equation (1) represents the line of best fit for the growth rate from the average Feed C and NaCl dataset presented in Figure 1. This osmolality change is responsible for a 50% reduction in the maximum proliferation rate.

(1) $${µ}_{max}=\;-1.4\times 10^{-4\;}\;Osm+0.085$$

This function is only linear in the examined range of osmolality, as decreasing the osmolality below 320 mOsm kg^−1^ may lead to a reduced specific growth rate as a result of lower nutrient abundance. The linear relationship is in line with other NaCl-spiked CHO cell cultures, where a slope of −9.3 × 10^−5^ was observed in comparison to −1.1 × 10^−4^ in this study [25,31].

**Figure 2:**
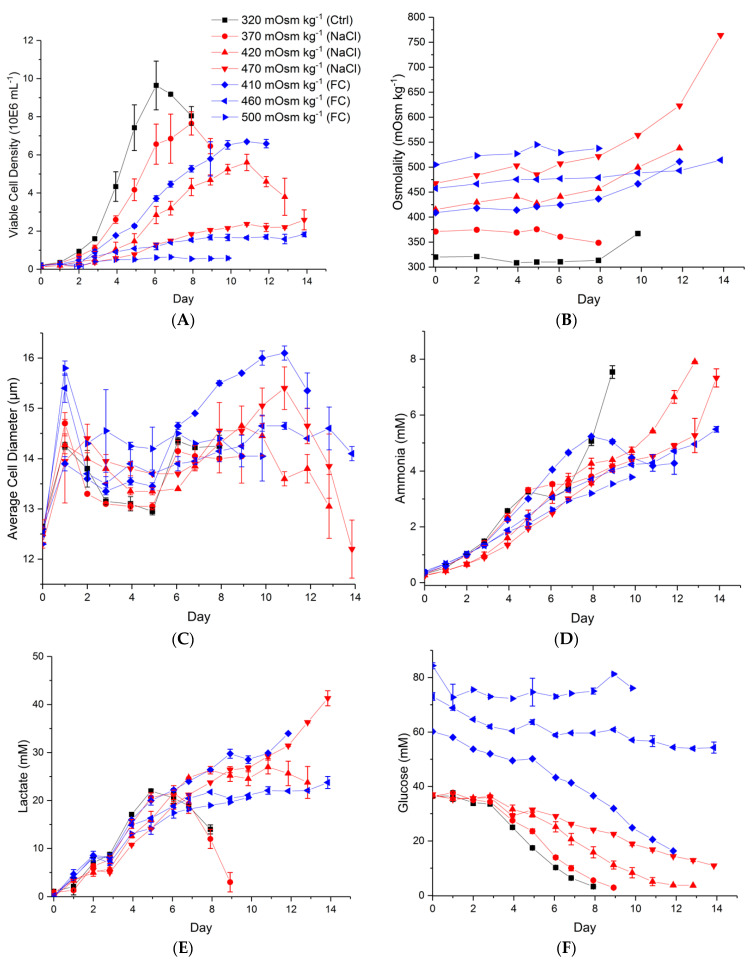

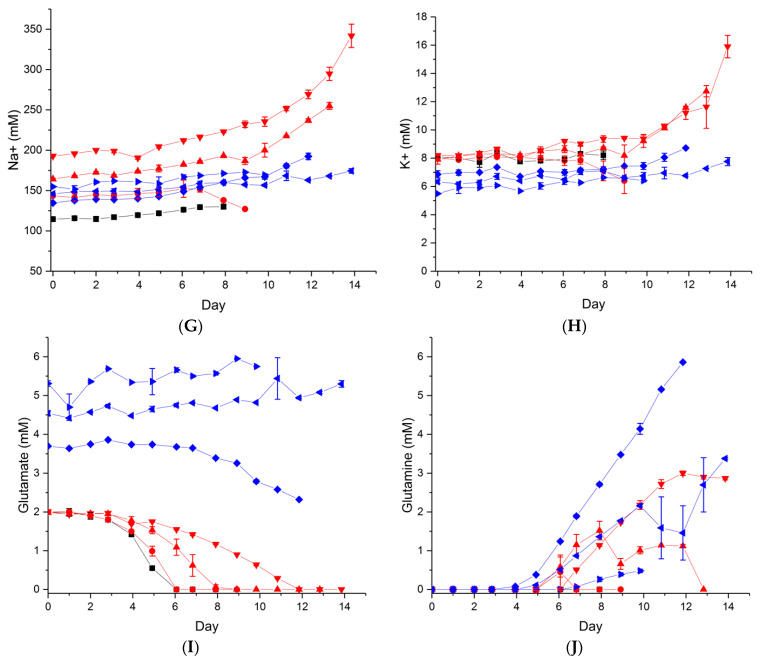
Time evolution of cell culture parameters under different osmolality conditions. (**A**) Viable cell density; (**B**) Osmolality value; (**C**) Average cell diameter; (**D**) Extracellular ammonia concentration; (**E**) Extracellular lactate concentration; (**F**) Extracellular glucose concentration; (**G**) Extracellular Na^+^ concentration; (**H**) Extracellular K^+^ concentration; (**I**) Extracellular glutamate concentration; (**J**) Extracellular glutamine concentration. Error bars represent the standard deviation of two biological replicates, except for the average cell diameter, which shows standard errors.

The viable cell density profiles corresponding to these experiments are shown in Figure 2A. There is a clear reduction in growth rate as osmolality increases by either salt or feed addition. These findings show how the addition of unnecessary feed can severely inhibit cell growth, as seen clearly in the case of 500 mOsm kg^−1^ (Feed C addition). The particular feed used in this study is typically added at 10% *v*/*v* in every other day of culture, starting on day 2 (based on the manufacturer’s instructions), but herein the feed was added on day 0. The maximum cell density is higher for the control in Figure 2A, which contradicts other published works about the effect of a spiked medium on a batch CHO cell culture’s performance [32,33]. The discrepancy in maximum cell density might be because the added feed in these published experiments is less than our minimum Feed C addition, i.e., 9% and 12% (*v*/*v*), respectively. Moreover, Feed C is commonly used in fed-batch cultures to prolong culture viability, whereas some of the previously reported spiked medium experiments were designed to boost productivity in batch cultures [34]. The osmolality value increases during culture (Figure 2B) mainly due to the accumulation of extracellular lactate, Na^+^ and K^+^ (Figure 2, panels E, G and H, respectively).

The average cell diameter is positively correlated to osmolality up to day 5 (Figure 2C), with its value increasing sharply 24 h post-inoculation, particularly for the Feed C-supplemented cultures beyond 410 mOsm kg^−1^. This phenomenon is known as Regulatory Volume Increase and is noticeable when compared to the control [35]. However, the trend changes from day 6 onwards, when key nutrients such as glucose and glutamate begin to be depleted from the control and NaCl-supplemented cultures (Figure 2F,I).

The ammonia concentration (Figure 2D) shows a gradual increase, but only three conditions exceed the 5 mM threshold, beyond which there is significant growth inhibition in this cell line [36]. However, in all cases, this threshold was reached during the stationary phase or late stage culture and was likely not detrimental to achieving peak cell density. Figure 2E,F show the extracellular lactate and glucose profiles, respectively, where the shift from production to consumption of the former is only observed for the control and 320 mOsm kg^−1^ conditions. The remaining cultures exhibited sustained lactate production throughout the culture, which, in some cases of NaCl-supplemented cultures, appears to be coming from sources other than glucose. The shift from lactate production to consumption is believed to be dependent on culture pH and the intracellular redox state, which was not measured herein [37].

The concentrations of Na^+^ and K^+^ remain constant for most cases except the two higher osmolality cases of NaCl addition, which show a further increase in the last days of culture. This increase in K^+^ is in alignment with the steep cell size decrease of the 420 and 470 mOsm kg^−1^ cases, which could mean that the cells are undergoing apoptosis. Apoptotic shrinkage is associated with isosmotic cell shrinkage as a result of K^+^ loss [38]. It has also been reported that elevated osmolality is associated with an increased apoptosis rate [39,40], although early apoptosis markers were not examined in this study. Glutamate (Figure 2I) was taken up by the cells, and some of it is hypothesised to have been channelled towards glutamine production (Figure 2J).

Proline and glycine betaine are known to act as osmoprotectants in mammalian cell culture [41]. Spent media analysis has indicated that residual proline concentrations are at least 60% of the initial concentration in CD CHO basal medium (data not shown). Proline was therefore in abundance until harvest in all cultures.

**Figure 3:**
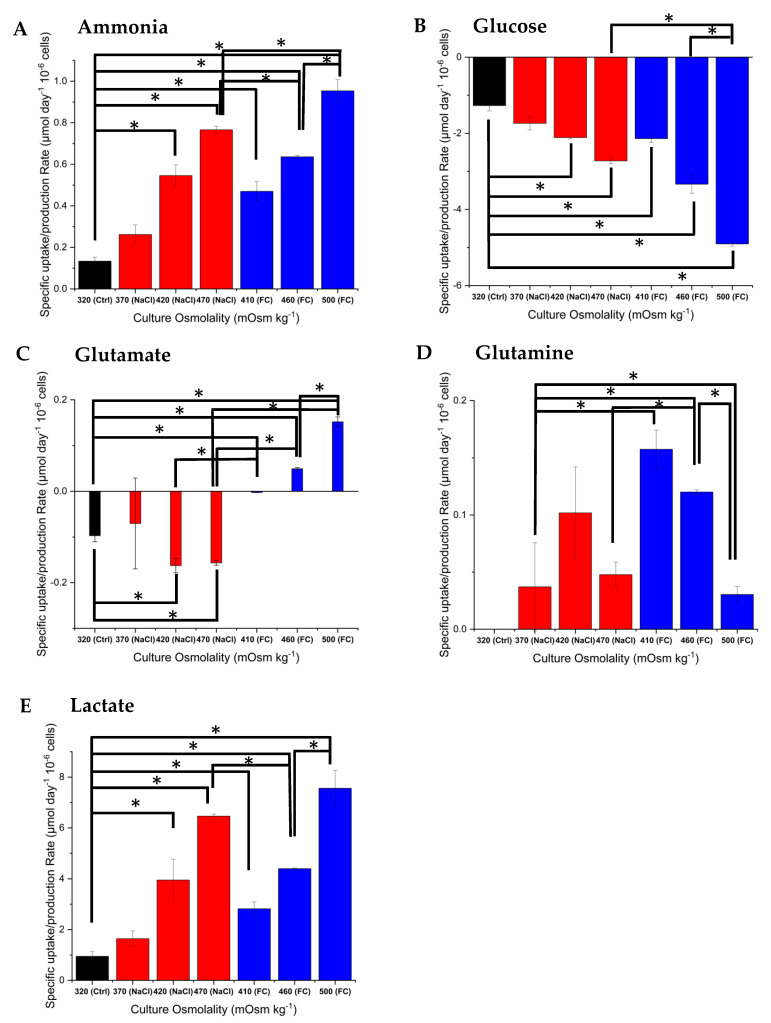
Specific uptake/production rates of (**A**) ammonia, (**B**) glucose, (**C**) glutamate, (**D**) glutamine and (**E**) lactate under different osmolality conditions. The control cultures produced no detectable levels of glutamine. Error bars represent the standard deviation of two biological replicates. For the analysis of significance (* *p <* 0.05), equal variance of each variable between different experiments was assumed.

Figure 3 shows the specific production and consumption rates of key metabolites for the exponential growth phase (i.e., calculated based on data from day 0 to day 6), during which we observed constant osmolality values. All cultures had higher specific glucose consumption rates compared to the control despite exhibiting lower specific cell growth rates. Regardless of the hyperosmolality treatment followed, the yield of lactate on glucose is higher as osmolality increases, pointing to inefficient carbon metabolism regardless of the induction method. It has been previously reported that the yield of lactate on glucose is increased at higher residual glucose concentrations [42]. Indeed, our experimental results point to the same phenotype. Comparing the residual glucose concentrations among cultures with the same induction method (Figure 2F), we see that the higher the glucose concentration, the higher the production rate of lactate (Figure 3E) during the cell growth period (initial adaptation period of two days followed by exponential cell growth). This behaviour continues during late stage culture, with the two cultures under normal osmolality conditions (control and 370 mOsm kg^−1^ NaCl) exhibiting a rapid decline in lactate concentration due to a reinternalisation of the metabolite by the cells as a carbon source (Figure 2E). In the case of the highest osmolality culture to which NaCl was added, a proportion of produced lactate appears to be coming from sources other than glycolysis. It has been previously reported that glutaminolysis can supply carbon and nitrogen in cancer cells, which are known to share similar metabolic features with CHO cells [43].

Interestingly, at osmolality values in the 410–420 mOsm kg^−1^ range, there is no significant difference in the ammonia production rate under the two hyperosmolality induction strategies. However, this changes in the 460–470 mOsm kg^−1^ range, in which NaCl cultures exhibit a significantly higher ammonia production rate (*p* < 0.05). Specifically, NaCl-supplemented cultures secrete more ammonia (Figure 3A) than Feed C-supplemented experiments. This is in line with the respective glutamine production rates, with the latter cultures using ammonia to synthesise and secrete glutamine at a higher rate than the former (Figure 3D). However, glutamate uptake/production rates do not follow the same pattern, with NaCl-supplemented cultures in the 460–470 mOsm kg^−1^ range consuming glutamate and Feed C-supplemented cultures secreting glutamate. Given that Feed C is rich in amino acids, we hypothesise that these cultures convert other amino acids to glutamate, which is then used to synthesise glutamine. Interestingly, the cultures at 500 mOsm kg^−1^ behave differently, showing increased ammonia and glutamate productivity and reduced glutamine secretion, pointing to different metabolic wiring under these high osmolality, nutrient-rich conditions.

### 2.2. Effect of Hyperosmolality on Titre and Specific Antibody Productivity and Glycosylation

**Figure 4:**
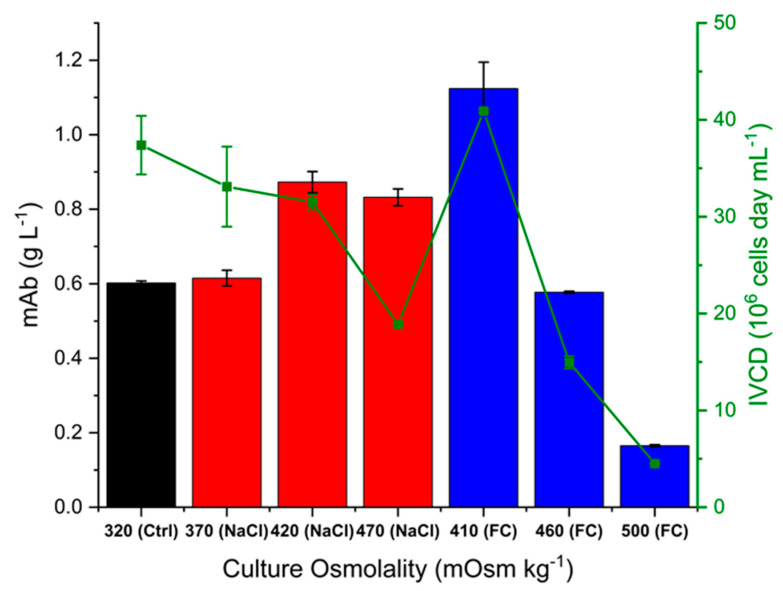
Final mAb titre (bars) and integral viable cell density (green points) for control, NaCl and Feed C experiments. Error bars represent the standard deviation of two biological replicates.

**Figure 5:**
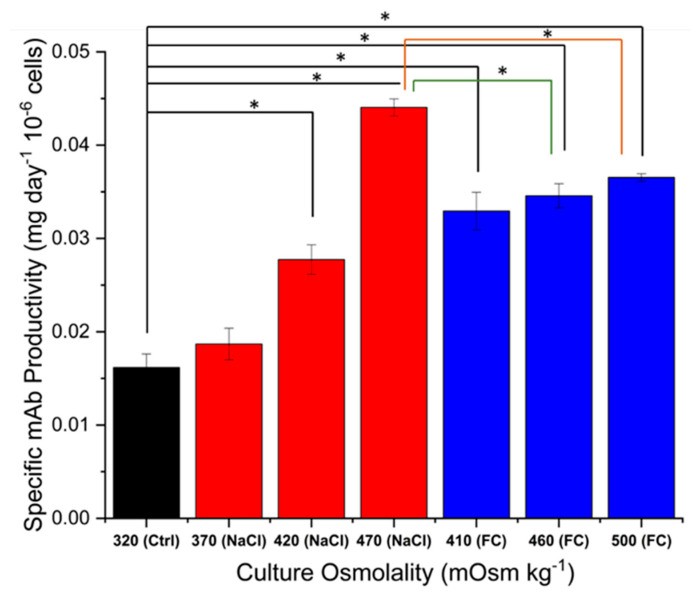
Specific antibody productivity for control, NaCl and Feed C experiments. Error bars represent the standard deviation of two biological replicates. For the analysis of significance (* *p* < 0.05), equal variance of each variable between different experiments was assumed.

Figure 4 shows the mAb titre at harvest for all cultures and the respective integral viable cell density (IVCD) values. The highest titre was obtained at 410 mOsm kg^−1^, achieved with Feed C addition, which also displayed the highest IVCD, although the latter was not significantly different from that of the control cultures. At the other end, the 500 mOsm kg^−1^ Feed C cultures had the lowest IVCD and mAb titre. Overall, the titre broadly follows the same trend as the IVCD, with the 470 mOsm kg^−1^ NaCl cultures being the exception. Looking at the specific mAb productivity values in Figure 5, we can see that the 470 mOsm kg^−1^ NaCl cultures had the highest rate of mAb secretion out of all conditions. This is not only higher with respect to the control cultures, but also significantly higher than for all Feed C-supplemented cultures. Interestingly, cultures supplemented with NaCl show an increase in specific mAb productivity that is positively correlated with the value of osmolality, whereas the Feed C-supplemented cultures show no statistically significant difference among the three different osmolality values. This potentially stems from the fact that the latter cultures utilised nutrients less efficiently due to the high levels of glucose abundance. Additionally, the 470 mOsm kg^−1^ NaCl and 410 mOsm kg^−1^ Feed C cultures exhibited a noticeably larger cell diameter after day 6, as seen in Figure 2C. This observation could point to expanded organelles supporting mAb synthesis (e.g., ER and Golgi apparatus), which is manifested, in turn, with a higher specific productivity. Overall, our findings are in broad agreement with the approaches that were reviewed by O’Callaghan and James to enhance mAb production [44].

**Figure 6:**
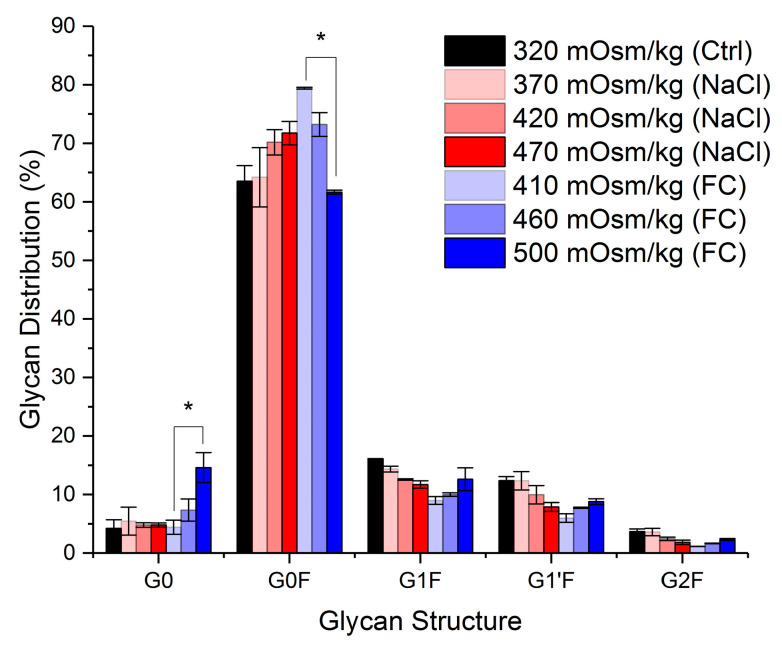
Glycosylation profile for control, NaCl and Feed C experiments at harvest. Error bars represent the standard deviation of two biological replicates. For the analysis of significance (* *p* < 0.05), equal variance of each variable between different experiments was assumed.

The effect of the two hyperosmolality induction methods on mAb glycosylation at harvest is illustrated in Figure 6. In the cultures supplemented with NaCl, there is no statistically significant difference in the glycan distribution (*p* > 0.05). However, cultures supplemented with increased amounts of Feed C showed a decline in G0F with increased osmolality values (18% difference between 410 and 500 mOsm kg^−1^). This was accompanied by an increase in G0 species (10% difference between 410 and 500 mOsm kg^−1^) and smaller increases in the percentage of galactosylated structures (G1F, G1′F and G2F). Lower levels of core-fucosylation and increased galactosylation, achieved either through genetic modifications [45] or alternative feeding regimes [46,47,48], are desired attributes in mAb-glycosylation that have been found to enhance the activity and efficacy of the glycoprotein [45,49]. Given that changes are manifested in both under-processed (G0) and more mature (G1F, G1′F and G2F) structures, it is difficult to pinpoint an underlying mechanism. However, it is unsurprising that the cultures exhibiting the highest specific productivity (410 mOsm kg^−1^ Feed C) also show an increased percentage of non-galactosylated structures. Given the high availability of nutrients in these cultures, it is likely that the intracellular availability of nucleotide sugars, the co-substrates for glycosylation, was not limiting. The high rate of antibody synthesis and secretion, on the other hand, may have limited antibody residence time in the Golgi, therefore reducing the extent of further glycan processing by b-1,4-galactosyltransferase (b4GalT), which is responsible for mAb galactosylation [50]. In contrast to the expected elevated distribution of non-galactosylated structures, core-fucosylation shows an unexpected behaviour among the cultures that include the addition of Feed C for osmolality regulation. As shown in Figure 6, the highest osmolality condition (500 mOsm kg^−1^ Feed C) has significantly lower levels of core fucosylation than the 410 mOsm kg^−1^ Feed C samples (*p* < 0.05), despite having a lower specific antibody productivity. The aforementioned observation could indicate the inhibition of a-1,6-fucosyltransferase (FucT) enzymatic activity or reduced gene (Fut8) expression due to higher osmolality levels, assuming that the levels of GDP-fucose, the co-substrate of core fucosylation, are unaffected by the osmolality changes.

Several process parameters, such as culture pH, temperature and ammonia levels, have been found to considerably affect recombinant protein glycosylation [51,52,53,54,55]. In principle, elevated ammonia levels result in increased intracellular pH conditions that inhibit b4GalT activity and expression, and consequently lead to reduced protein galactosylation [56,57,58]. However, the cultures with osmolality levels of 320 mOsm kg^−1^, 420 mOsm kg^−1^ and 470 mOsm kg^−1^ that presented the highest ammonia concentrations at harvest (Figure 2) did not collectively demonstrate any significant reduction in IgG galactosylation (Figure 6). It is important to note that harvest samples correspond to different culture days, which is known to influence the glycan structures obtained [59]. Typically, the glycosomal shifts to a higher abundance of under-processed structures as the culture progresses, which could partly explain the differences between the 500 mOsm kg^−1^ Feed C cultures, harvested on day 10, and the 410 mOsm kg^−1^ Feed C cultures, harvested on day 12. As the cultures were all harvested at 80% culture viability, the observed differences are not attributable to an accumulation of intracellular material from lysed cells. Interestingly, the results for the 500 mOsm kg^−1^ Feed C cultures are broadly aligned with the control culture data (harvested on day 8).

The two culture conditions under which cells exhibited the largest cell size increase, i.e., 410 mOsm kg^−1^ (Feed C) and 470 mOsm kg^−1^ (NaCl), had a relatively high specific productivity, but the former also had a higher proliferation rate. It is obvious that not all larger cells are high producers, but some of the high producers show a large volume phenotype (i.e., 15.7 μm and 14 pg/cell/day, in comparison to less than 14.3 μm and 3.2 pg/cell/day for low producing cells) [60]. The larger cell volume could indicate an increase in the volume of cellular organelles such as the ER, which would indicate an increased protein synthesis and folding capacity and, thus, a higher protein secretion rate under hyperosmolar conditions [60].

### 2.3. Effect of Hyperosmolality on Cell Volume

Having explored the effect of hyperosmolality induced by feed and salt addition in the beginning of the CHO cell cultivation in flasks, we then looked at a set of typical fed-batch CHO cell cultures in bioreactors. The latter are better representatives of the typical production conditions, under which extracellular osmolality increases up to 70% above the initial value because of the feeding and excretion of metabolites, which has been previously reported to lead to an increase of up to four times in cell volume [2,61]. Additionally, in the bioreactor environment we could effectively control the culture’s pH, but also explore the effect of shifting the temperature to mild hypothermic conditions during exponential growth, which is a commonly employed strategy to prolong culture duration and achieve increased product titre [52].

**Figure 7:**
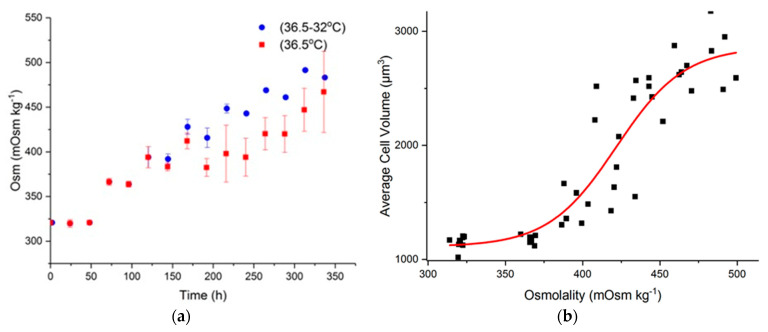
(**a**) Osmolality profile for the bioreactor runs at 36.5 °C (blue) and with a temperature shift to 32° C on day 5; (**b**) Average cell volume against culture osmolality for both bioreactor datasets.

In our bioreactor study, metabolism was more active at 36.5 °C, with both glucose and lactate being consumed more readily than under mild hypothermia [62]. This led to a slower increase in osmolality at 36.5 °C for the same feeding schedule (Figure 7a). Plotting the average cell size as a function of osmolality illustrates the effect of osmolality on cell volume (Figure 7b). Outliers in cell size, such as the cell aggregates and fragments observed particularly in late stage culture, were removed from the data, limiting the diameter range to between 10 and 20 µm based on previous observations [3,63].

**Figure 8:**
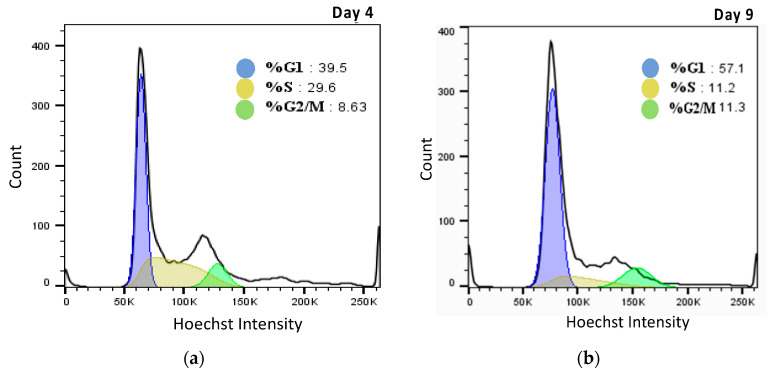
(**a**) Cell cycle distribution on day 4 of culture; (**b**) Cell cycle distribution on day 9 of culture as determined by a Flowtop multicycle DNA analysis.

**Figure A2:**
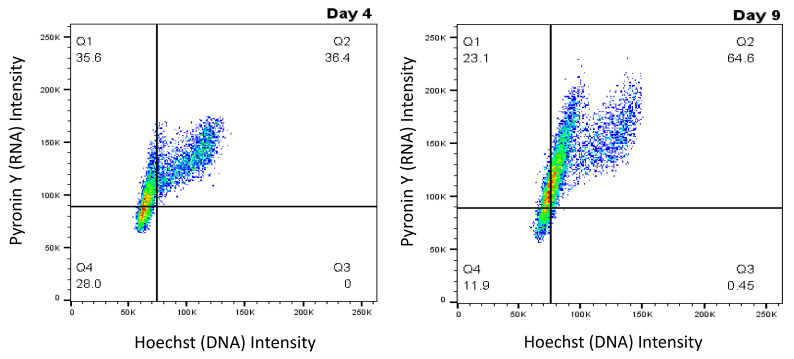
RNA (y axis) and DNA (x axis) distribution on days 4 and 9 of the bioreactor run at physiological temperature.

Although there appears to be a correlation between osmolality and cell volume, this is not sufficient to fully describe the data presented herein. For example, even though the largest cell size is observed at the end of culture, this did not correspond to the highest osmolality value, pointing to a potential threshold for the osmolality effect on cell volume. A secondary factor that could impact cell volume is cell cycle distribution, which also changes during culture. Two samples from the exponential phase (day 4) and stationary phase (day 9) from the bioreactors run at 36.5 °C were analysed by flow cytometry, as shown in Figure 8. An increase in the proportion of cells in the G_2_/M phase was observed as the culture progressed, in parallel to an increase of the cells in the G_1_ phase (RNA content results are shown in Appendix A Figure A2).

The percentage of cells in G_0_/G_1_ is known to be the highest throughout the culture duration and usually increases in late stage culture [4]. This has led to the assumption that cells are quiescent, arrested in G_0_ [64,65]. Distinguishing cells in G_1_ from those in G_0_ is important, as the former is more metabolically active than the latter [66].

**Figure A3:**
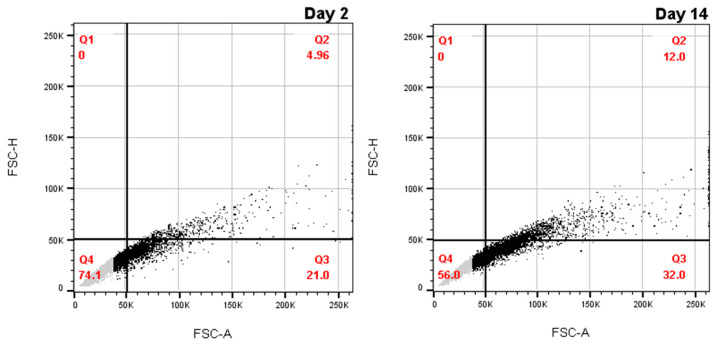
Forward scatter height versus area for single cells on days 2 and 14 from the bioreactor run at physiological temperature.

The shift in cell volume in the bioreactor was also shown by analysing two samples from days 2 and 14 of the bioreactor samples (Figure A3). These figures show the difference in cell size of early and late days of cell culture. The increase in cell size is clearly shown where the smallest cell population in Q4 dropped from 74% to 56% and caused an increase in larger cell quarters Q2 and Q3. More importantly, the cell cycle distribution and increase in the G_2_/M phase at the later stage of culture likely contributes to the increase in average cell volume during the bioreactor runs beside the effect of osmolality.

In late stage culture we see a decrease in the average cell diameter. Regulatory Volume Decrease (RVD) is an adaptive mechanism achieved by the extrusion of intracellular osmotically active solutes up to reach the water balance imposed by the new condition. The osmolytes involved are the main intracellular ions K^+^ and Cl^−^ and several small molecules, including amino acids, polyalcohol and amines [67]. Another contributing factor is expected to be the increase in apoptotic cells, which would lead to cell shrinkage [38]. Cell cycle distribution also contributes to the overall cell volume increase during culture based on the increase in the cell subpopulation in the G_2_/M phase seen in late stage culture.

Takagi et al. evaluated the effect of osmolarity on CHO cell growth rate, glucose consumption, lactate production and tepa productivity in adhesion and suspension cell culture [31]. The critical osmolality value was 450 mOsm kg^−1^ for suspension culture and lower for adhesive cell culture. Their average reported cell diameter varies from 7.25 to 9.14 µm, which is smaller than the typical diameter values for CHO cells [3].

## 3. Materials and Methods

### 3.1. Cell Line Maintenance

CHO GS46 producing a chimeric IgG_4_ antibody (Lonza Biologics) was revived and cultured in shake flasks (Corning, NY, USA) in CD CHO medium (Life Technologies, Paisley, UK) and shaken at 140 RPM. The cells were subcultured in fresh medium on day 3 after revival and every four days, subsequently, at a seeding density of 2 × 10^5^ cells mL^−1^. The first and the second passages were supplemented with the selection agent L-Methionine Sulfoximine (MSX, Sigma-Aldrich, Dorset, UK) at a concentration of 25 µM.

### 3.2. Bioreactor Experiments

The bioreactor osmolality data presented herein were obtained from four bioreactor experiments, described in Goey et al. (2017). Briefly, the bioreactors (Applikon Biotechnology, Schiedam, the Netherlands) were inoculated at a seeding density of 3  ×  10^5^ cells mL^−1^ with an initial cell culture volume of 1.2 L. The temperature, pH and dissolved oxygen were controlled at 36.5 °C, 7.0 and 50%, respectively. All vessels were supplemented with CD EfficientFeed™ C AGT™ at 10% *v*/*v* on alternate days, starting on day 2. In two of the four vessels, the temperature was reduced to 32 °C on day 5 of culture.

### 3.3. Flask-Based Osmolality Experiments

A total of 14 cultures with a working volume of 55 mL in 250 mL Erlenmeyer flasks were carried out using CD CHO medium (ThermoFisher Scientific, Paisley, UK). Two of these were not supplemented with any osmolyte and used as a control. The osmolality of the culture medium was increased before inoculation from 320 (control condition) to three values up to 500 mOsm kg^−1^ by addition of 5 M of NaCl (9200 mOsm kg^−1^) or CD EfficientFeed™ C AGT™ (1130 mOsm kg^−1^) (ThermoFisher Scientific, Paisley, UK). The added volume to increase osmolality is always less than 4% of the initial volume, making any dilution effect negligible. Cultures were seeded at 2 × 10^5^ cells mL^−1^. Duplicate flasks for each condition were placed at 37 °C and 5% CO_2_ and shaken at 140 RPM. Samples were withdrawn daily for further analysis. Cultures were harvested when culture viability dropped below 80%.

### 3.4. Analytical Methods

All analyses were carried out for two biological replicates and two technical replicates. Cell volume, cell density and culture viability were measured daily with a Nucleocounter NC-250 (ChemoMetec, Lillerød, Denmark). BioProfile FLEX (Nova Biomedical, Waltham, MA, USA) was used to perform automated enzymatic assays to determine the extracellular concentrations of glucose, lactate, glutamine and glutamate, while the ammonia concentration was measured by electrochemical means with a phosphate assay.

The antibody titre was measured on the last day of the culture by biolayer interferometry using the Blitz system (Pall ForteBio Europe, Westborough, MA, USA). The osmolality of supernatant samples was measured by Osmomat 3000 (Gonotec, Berlin, Germany). The equipment was calibrated with 0, 300 and 800 mOsm kg^−1^ solutions, and sample sizes were 50 µL in 500 µL measuring vessels (Gonotec, Berlin, Germany).

### 3.5. Flow Cytometry

Flow cytometry analysis was carried out on two bioreactor samples to determine the cell cycle distribution. In this experiment, a double staining procedure was followed [29,30]. Hoechst 33342 and Pyronin Y were used to stain the DNA and RNA, respectively, of two samples. The first one represents the day 4 population, and the second represents the day 9 population of the bioreactor operated at physiological temperature. This choice is based on our previous knowledge of the cell cycle distribution of the CHO cell culture, which differs in between these two days [31]. PBS buffer was supplemented with 0.5% (*w*/*v*) bovine serum albumin and 1 mM EDTA. Hoechst 33342 and Pyronin Y were added to the fluorescence-activated cell sorting, FACS, at a concentration of 2 and 4 µg/mL, respectively, and kept at 4 °C in the dark before use.

1 × 10^6^ cells were harvested by centrifugation for 5 min at 200× *g* at room temperature and washed with 10 mL PBS. Cells were then resuspended in 0.5 mL PBS and fixed by dropwise addition of 4.5 mL pre-chilled 70% ethanol at −20 °C. The sample was vortexed and kept at −20 °C for 2 h. The ethanol was removed by centrifugation at 300× *g* for 3 min at room temperature. Cells were then washed twice with 5 mL FACS buffer with a centrifugation step for 5 min at 200× g each time. 0.5 mL of the prepared staining solution was added to the cells and incubated for 20 min at room temperature. Fluorescence was analysed by BD LSR Fortessa (BD Biosciences, San Jose, CA, USA). UV (355 nm) and blue (488 nm) lasers were set to analyse the samples. 450/50-nm and 575/26-nm band passes were chosen for Hoechst 33342 and Pyronin Y, respectively, with linear acquisition [32]. Debris and doublets were excluded by gating on forward scatter and side scatter plots. The removal of cell fragments and clumps was performed as the first step in the flow cytometry sample. This was achieved by using a pulse geometry gate such as FSC-H against FSC-A to focus on a single cell population.

### 3.6. Glycan Analysis

Prior to glycan analysis, 0.5 mL of cell culture supernatant was filtered with 0.22 μm microcentrifuge filters (Sigma Aldrich, Dorset, UK) and incubated with Protein A agarose beads (Sigma Aldrich, Dorset, UK) for 2 h at room temperature. After the incubation was complete, the IgG_4_ was eluted using 0.2 M Glycine pH 2.5 solution. Following elution, the IgG_4_ was transferred to 1× PBS (Gibco, Thermo Fisher Scientific, Waltham, MA, USA) through buffer exchange, using 30 KDa cut-off micro-centrifuge filters (ThermoFisher Scientific, Paisley, UK). For glycan analysis, the C100HT Glycan analysis kit was used (SCIEX, Brea, CA, USA). Briefly, 100 μg IgG_4_ was denaturated at 60 °C for 8 min, using the kit’s denaturation solution. Following denaturation, the glycans were digested using 500 units, per sample, of the PNGase F enzyme (New England Biolabs, Hertfordshire, UK) for 20 min and at 60 °C. Released glycans were labelled with APTS-solution at 60 ℃ and for 20 min. Labelled glycans were washed three times with acetonitrile (Sigma Aldrich, Dorset, UK), subsequently eluted in water and loaded to a C100HT Glycan analysis–capillary electrophoresis (SCIEX, Brea, CA, USA) instrument for glycans analysis.

### 3.7. Statistical Analysis

Uptake and secretion rates were calculated for each replicate before calculating reported average values. A student t-test was used to determine the hyperosmolality significance on reducing the growth rate and the significance of the difference between the NaCl and Feed C treatments. Two sample t-tests (*p* < 0.05) with equal variance for each variable were employed for the evaluation of the glycan analysis. Origin 2020 (OriginLab, Northampton, MA, USA) was used for the statistical analysis of IgG glycosylation.

## 4. Conclusions

In this study we examined the effect of hyperosmolar conditions on CHO cell growth, cell volume and antibody production. We compared two methods for inducing hyperosmolality, addition of NaCl and supplementation of Feed C, a commercial feed designed to extend cell growth. The extent to which osmolality affects cell growth and antibody titre varied significantly and depended on the value of osmolality as well as the method used to increase it. This is because, although hyperosmolar conditions increased the specific antibody productivity across all conditions, they also adversely affected growth, meaning that the total titre was lower than the control in certain cases. Surprisingly, cultures supplemented with NaCl exhibited a more consistent behaviour compared to those supplemented with Feed C, pointing to the negative effect of overfeeding on growth rate.

Increased osmolality led to an increase in specific antibody productivity, although not as high as reported in previous studies. The supplementation of NaCl did not alter the antibody glycosylation profile significantly. In contrast, hyperosmolar conditions induced by Feed addition resulted in a desirable shift from G0F to both non-cacodylate (G0) and cacodylate-galactosylated (G1F, G1′F and G2F) structures, with the extent of the shift appearing to correlate with the osmolality value.

At high osmolality values, cell metabolism appears to be less efficient. Hyperosmolality slowed down growth, which is useful for prolonging cell culture duration. Undoubtedly, the osmolality upshift would yield a better titre if the addition took place at a later culture stage, when the viable cell density had already reached a plateau, but process optimisation was not the aim of this study. Osmolality was also shown to be a significant determinant of cell volume, even though the largest cell size at the end of culture did not correspond to the highest initial osmolality value. This could point to a threshold for the osmolality effect on cell volume.

Overall, the effect of osmolality due to the addition of nutrient-rich feed supplements appears to be multifaceted. Although feed addition usually prolongs culture longevity and increases the specific productivity, and therefore the total antibody yield, it is associated with wasteful metabolism and may, in fact, hinder growth when leading to hyperosmolar conditions in early-stage culture. Under such conditions, it also led to a deterioration of the glycan distribution profile in this study. Platform antibody processes typically employ pre-formulated media and feeds, which undoubtedly speed up process development. However, our results indicate that overfeeding may, in fact, result in lower productive cell time (indicated by IVCD). Taken together with other recent studies, our findings point to the need for tailoring feed formulation and addition to the particular cell line and product at hand. Additionally, they point to the need for expanding the range of in-process analytics to amino acids, with a view to informing the feeding strategy in near real-time.

## Figures and Tables

**Figure A1:**
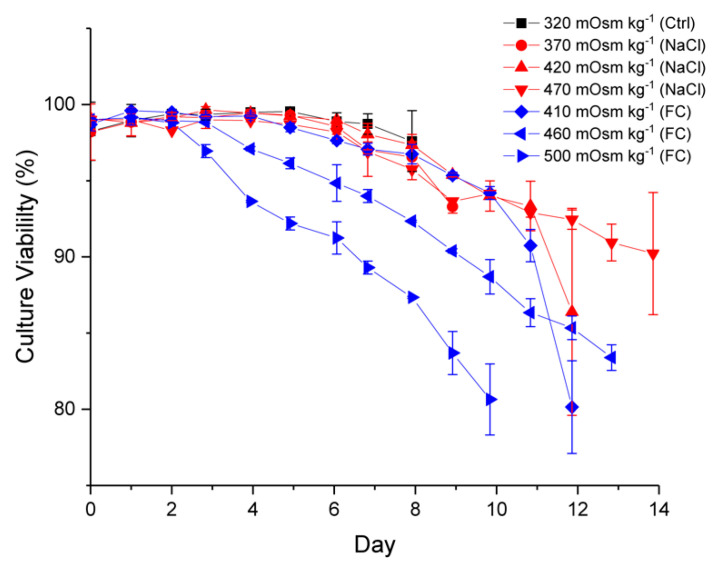
Culture viability profile across different osmolality values. The cultures were harvested on the day after the final measurement reported herein, when the viability in one of the biological duplicates dropped below 80%.

## Data Availability

Data can be made available upon request.

## Appendix A

Growth and uptake/secretion rate calculation.

To calculate the growth rate:

$$\mu =\frac{ln\frac{x_{1}}{x_{0}}}{t_{1}-t_{0}}$$

μ (h^−1^) is growth rate in
$x_{1}$ (10^6^ cell mL^−1^) is viable cell density at time $t_{1}$

To calculate the specific substrate/metabolite uptake/production rate:

$$q=\;\frac{C_{0}-C_{1}}{IVCD_{1}}$$

q (μmol day^−1^ 10^−6^ cell) is the metabolite (or mAb) uptake/secretion rate
$C_{0}$ (mM) is the initial substrate (or mAb) concentration and $C_{1}$ (mM) the concentration at $t_{1}$ (day 6)
$IVCD_{1}$ (10^6^ cell day mL^−1^) is the integral viable cell density at $t_{1}$ (day 6)
